# Risk Reversal of Oral, Pharyngeal and Oesophageal Cancers after Cessation of Betel Quid Users: A Systematic Review and Meta-Analysis

**DOI:** 10.5334/aogh.3643

**Published:** 2022-01-11

**Authors:** Ruchika Gupta, Lorena C. Mariano, Suzanne T. Nethan, Ashwini Kedar, Dhirendra N. Sinha, Saman Warnakulasuriya, Luis Monteiro, Shashi Sharma, Sanjay Gupta, Shalini Singh, Kurt Straif

**Affiliations:** 1 ICMR-National Institute of Cancer Prevention and Research, I-7, Sector-39, Noida, IN; 2 UNIPRO, Oral Pathology and Rehabilitation Research Unit, University Institute of Health Sciences (IUCS), CESPU, Gandra, Portugal and Medicine and Oral Surgery Department, Instituto Universitário de Ciências da Saúde (IUCS), Gandra, PT; 3 School of Preventive Oncology, Patna, IN; 4 Program Officer QC Process, STOP C clinical trial, YRGCARE, Delhi, IN; 5 Faculty of Dentistry, Oral & Craniofacial Sciences, King’s College London, WHO Collaborating Centre for Oral Cancer, London, United Kingdom, GB; 6 Independent researcher, IN; 7 WHO FCTC Global Knowledge Hub on Smokeless Tobacco, ICMR-National Institute of Cancer Prevention and Research, I-7, Sector-39, Noida, IN; 8 ISGlobal, Barcelona, Spain, and Boston College, Chestnut Hill, MA, USA

## Abstract

**Background:**

Areca nut (AN), the principal ingredient of betel quid (BQ) has been categorized as a human carcinogen associated with various cancers of upper aerodigestive tract. However, there has been no attempt at summarizing the risk reversal of oral and other cancers after cessation of BQ with or without tobacco (BQ+T/BQ-T).

**Objective:**

To analyze the effect of cessation of betel quid without tobacco (BQ-T) and with tobacco (BQ+T) on reversal of the risk of oral, pharyngeal and oesophageal cancers.

**Methods:**

A systematic literature search was conducted for publications evaluating risk of these three cancers among current and former users of BQ-T or BQ+T. The overall as well as subgroup meta-relative risks (meta-RR) were estimated using random-effect models.

**Results:**

A total of 14 studies, seven each providing estimates for BQ-T and BQ+T, were identified. For BQ-T and oral cancer, a 28.9% risk reversal was observed among former users (meta-RR 5.61, 95% CI 2.24–14.04) compared to current users (meta-RR 7.89, 95% CI 3.90–15.98). A risk reversal of 48% was noted for pharyngeal cancer – former users (meta-RR 2.50, 95% CI 1.43–4.38), current users (meta-RR 4.81, 95% CI 2.05–11.30). For oesophageal cancer, no appreciable difference in risk was observed between current and former users.

For BQ+T and oral cancer the overall meta-RR indicated a higher risk in former than in current users. However, sensitivity analysis including only better-quality studies showed a modestly lower cancer risk in former than in current users. Compared to current users, the risk in former users who quit less than 10 years ago (meta-RR 1.21, 95% CI 0.90–1.63) was increased, but decreased in former users who quit more than 10 years ago (meta-RR 0.72, 95% CI 0.48–1.07).

**Conclusion:**

Our analysis highlights for the first time the potential of risk reversal for oral and pharyngeal cancers following cessation of BQ-T and for oral cancer in long-term quitters (greater than 10 years) of BQ+T. The suggestive evidence from this systematic review further supports the imperative need of a strong policy to reduce the initiation of BQ use and inclusion of interventions for BQ cessation in cancer control efforts especially in geographic regions where BQ chewing is prevalent.

## Introduction

Areca nut (AN), the fourth most commonly consumed addictive substance and the main ingredient of betel quid without added tobacco (BQ-T) or with tobacco (BQ+T), has been causatively linked to oral potentially malignant disorders, oral cancer, oesophageal cancer and – for BQ+T – pharyngeal cancer [1,2]. Further, since the latest International Agency for Research on Cancer (IARC) evaluations, a meta-analysis reported a statistically significant association between BQ-T and cancer of the oropharynx [3]. AN is consumed by about 600 million individuals worldwide, though the highest burden of use is reported from the Pacific Islands, South and South East Asia [4]. In these regions with high prevalence of AN consumption, the use of AN has a long religious and cultural tradition, often linked with beliefs about various beneficial effects. Despite the classification of AN, BQ+T and BQ-T as human carcinogens by the IARC [1,5], to our knowledge, there has been no attempt at summarizing the risk reversal of oral and other cancers after cessation of BQ-T or BQ+T. An evaluation of the effect of BQ-T and BQ+T cessation on cancer risk would provide evidence-based advice to support measures of cessation among current BQ users.

The present systematic review and meta-analysis (SRMA) was performed with an aim to identify, synthesize and meta-analyze, for the first time, the existing global literature on the risk of oral, pharyngeal and oesophageal cancers in current and former users of BQ-T/BQ+T. This is an effort to provide an estimate of the effects of BQ cessation on the risk of upper digestive tract cancers causally linked to AN use and inform cancer control programs in the concerned regions for inclusion of BQ cessation advice to the public and in their guidelines for healthcare workers.

## Materials & Methods

### Literature search

The primary studies included in the pertinent IARC monographs, particularly volumes 85 and 100E and including data on oral, pharyngeal or oesophageal cancer in users of BQ-T or BQ+T were considered [2,5]. In addition, a search was performed in the electronic databases (PubMed/Medline, ProQuest, Web of Science and Cochrane Library) from inception till August 31, 2021 using relevant combinations of search terms listed in Supplementary Table 1. Further, the reference list from each included study was manually screened by two authors independently (RG, KS) to identify potentially relevant studies.

### Eligibility criteria

Research articles published in English or those with available English translations were included in the analysis when original results of cancer of oral cavity and/or pharynx and/or oesophagus as the outcome variable, AN or BQ-T or BQ+T as the exposure variable with separate ORs/RRs for current users and former/ex-users of AN or BQ-T or BQ+T compared to non-users were reported or could be calculated from published data.

Reports with lack of clarity in the composition of the chewing product (BQ-T vs BQ+T); case series, case reports, letters or reviews; studies with potentially malignant disorders of the oral cavity and those exploring the cancer risk due to BQ-T/BQ+T but not providing risk estimates for former or ex-users were excluded from the SRMA.

### Data Extraction

From the included articles, three authors (RG, STN, KS) independently extracted the data on first author, year of publication, country of study, study population, sample size, sex, exposure (with distinction between BQ-T and BQ+T; definition of cessation) and outcome variable, risk estimates (with 95% confidence intervals) for current and former users and adjustment for potential confounders like tobacco smoking, alcohol drinking and other variables. For studies reporting on the same or overlapping study populations, results of the most informative study were included. Studies which did not present separate results for BQ-T vs BQ+T but reported a strong majority of BQ+T users among their study population were included in the meta-analyses on the cessation of BQ+T use. Preference was given to relative risks adjusted for the main potential confounders. Any instance of disparity in the results of the three authors was adjudicated by a senior author (SG).

### Statistical analysis

We used the software program Review Manager (RevMan) version 5.3 (Copenhagen: The Nordic Cochrane Centre, The Cochrane Collaboration, 2012) to perform all meta-analyses. Random-effect models were used since there was methodological diversity among the included studies. Substrata-specific relative risks of individual studies (e.g. by subsite of cancer or by categories by duration of cessation) were first combined at study level using fixed-effect meta-analytic methods. Where necessary, reported relative risks for current and former users were converted to non-users as the standard reference group for the meta-analyses. Only for the meta-analysis by duration of cessation current users had to be chosen as the reference group. Subgroup analyses (by sex) and sensitivity analyses by study characteristics (study design, definition of cessation, and adjustment for potential confounders) were performed. We performed Q tests and I^2^ tests to evaluate the heterogeneity, defining a significant heterogeneity as Cochrane Q < 0.10 and/or I^2^ > 50%. Sensitivity analyses were carried out by dropping studies to address potential confounders. For meta-analyses with low number of included studies we performed qualitative visual inspection of forest plots.

Since the objective of this SRMA was evaluation of change in cancer risk following cessation of BQ-T or BQ+T, the difference in risk ratio of current and former users, both in comparison with non-users, was calculated as a percentage and considered as an estimate of risk reversal after cessation of BQ-T or BQ+T, as depicted in the following equation:

Risk\ reversal = \frac{{\overline {es{t_f}} - \overline {es{t_c}} }}{{\overline {es{t_c}} }}\ {\times}\ 100

Where \overline {es{t_f}} is meta-RR of former BQ-T/BQ+T users and ***est_c_*** is meta-RR of current BQ-T/BQ+T users.

## Results

The initial search yielded a total of 620 publications, of which 22 duplicates were removed. After exclusion of irrelevant articles and those not fulfilling the inclusion criteria, 24 full texts were assessed for inclusion. Of these 10 were further excluded (nine for reporting only on cancers of other sites and one for including overlapping study population) (Supplementary Figure 1). Thus, the present SRMA included a total of 14 studies – seven studies including separate risk estimates for current and former users of BQ-T (four providing estimates for oral cancer [6,7,8,9], three for pharyngeal [8,9,10] and two for oesophageal cancer [11,12]) and seven reports of BQ+T cessation and oral cancer [13,14,15,16,17,18,19]. Two studies included both oral and pharyngeal cancers in the outcome variable [8,9], and hence, the number of studies included in the SRMA for BQ-T was seven. Only one study reported results on cessation of BQ+T in relation to cancers of the pharynx and oesophagus [14].

### I. BQ-T cessation and risk of cancer

Of the four studies on BQ-T and oral cancer, three were reported from Taiwan [6,8,9] and one from Papua New Guinea [7]. Two studies on pharyngeal cancer [8,9] and both reports on oesophageal cancer [11,12] were conducted in Taiwan while one study on pharyngeal cancer [10] was reported from southern China. All seven studies were case-control in design, Majority of the studies included only or predominantly male subjects. The duration of cessation of BQ-T for defining former or ex-users of BQ-T was more than one year in four studies [9,12,13,14], more than six months in one study [11] while two studies [8,10] did not mention this aspect in their reports. Tobacco smoking as a potential confounder was adjusted in all the studies while alcohol consumption was adjusted for in all except one [7]. The characteristics of the studies included in the SRMA are given in Supplementary Table 2.

#### Quantitative analysis

**Figure 1:**
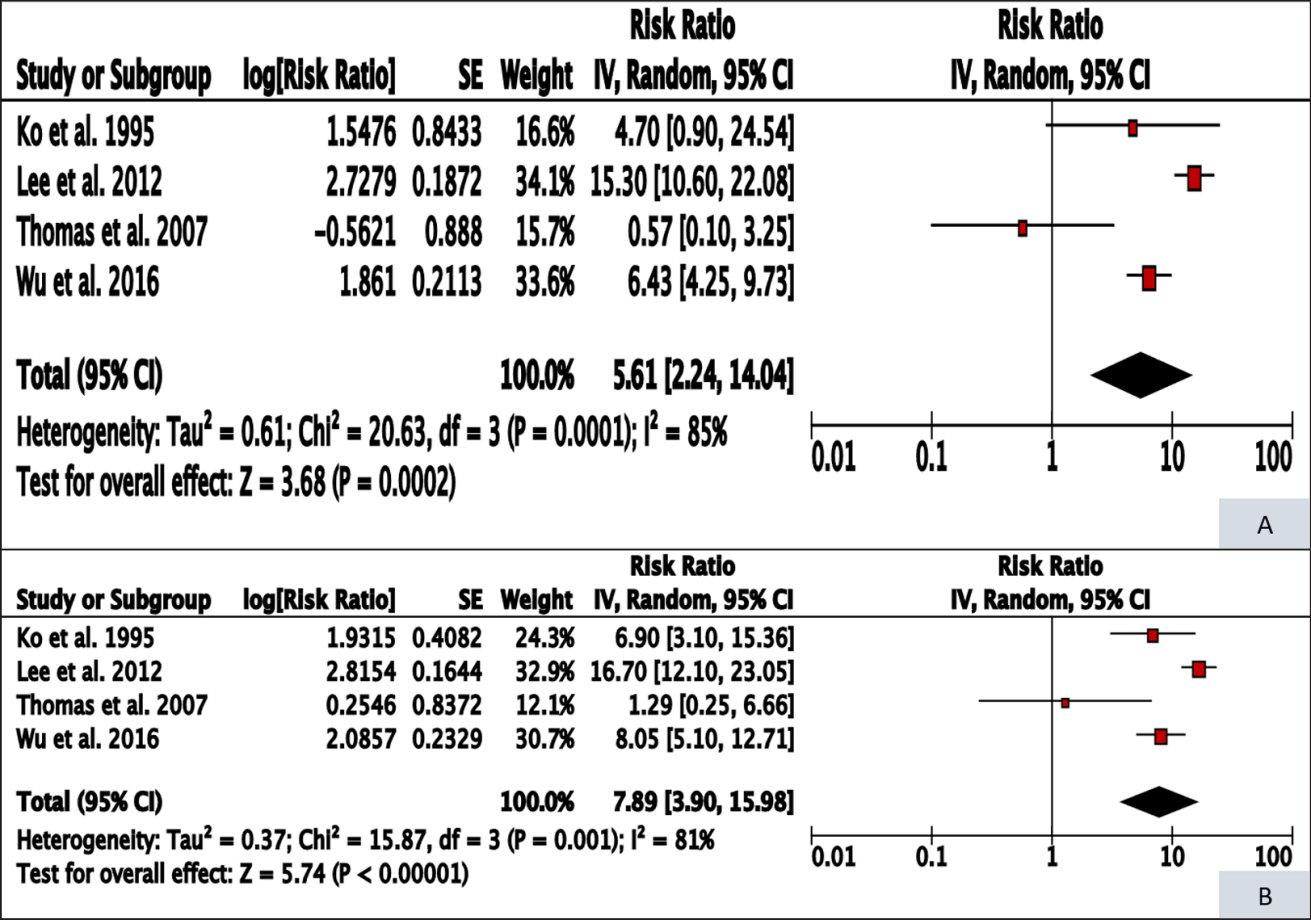
Forest plot and meta-RR (random effect) of the effect of BQ-T cessation on oral cancer risk (A – former users, B – current users).

Analysis of the four studies on BQ-T and oral cancer gave a meta-relative risk of former users (meta-RR 5.61, 95% CI 2.24–14.04) which was lower than that for current users (meta-RR 7.89, 95% CI 3.90–15.98), but 95% confidence intervals overlapped, as depicted in ***Table 1*** and ***Figure 1***. The difference represents a 28.9% reversal in oral cancer risk after cessation of BQ-T chewing. Sensitivity analysis including only those studies where confounder adjustment for tobacco smoking and alcohol consumption was done gave a meta-RR for current BQ-T users as 10.35 (95% CI 5.71–18.77) and former users as 8.95 (95% CI 4.21–19.0), giving a 13.5% risk reversal after BQ-T cessation (Supplementary Figure 2). Only one study reported results by duration since cessation with decreased risks after >20 years of cessation [9].

**Table 1:** Summary risk estimates of studies evaluating the risk of oral, pharyngeal or oesophageal cancer in current and former BQ-T users.


SITE OF CANCER	PRODUCT AND TYPE OF USERS	SUMMARY RISK RATIO	95% CI	EGGER’S/BEGG’S TEST FOR PUBLICATION BIAS (P VALUE)

**Oral cancer**	Current BQ-T user	7.89	3.90–15.98	0.02

	Former BQ-T user	5.61	2.24–14.04	

**Pharyngeal cancer**	Current BQ-T user	4.81	2.05–11.30	1.00

	Former BQ-T user	2.50	1.43–4.38	

**Oesophageal cancer**	Current BQ-T user	2.42	1.25–4.69	0.81

	Former BQ-T user	2.64	1.87–3.72	


**Figure 2:**
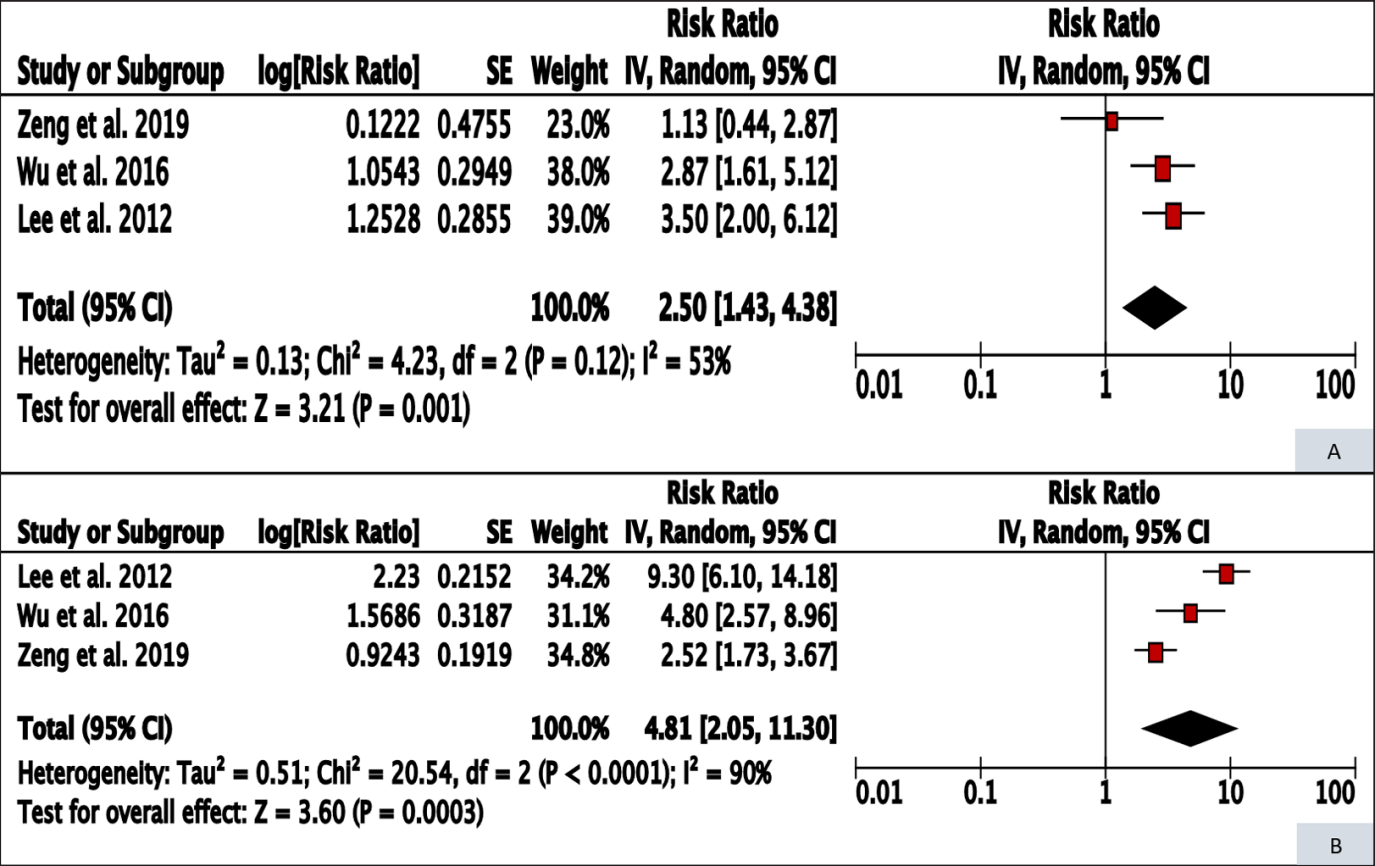
Forest plot and meta-RR (random effect) of the effect of BQ-T cessation on pharyngeal cancer risk (A – former users, B – current users).

The three studies on BQ-T and pharyngeal cancer gave a summary risk ratio of 2.50 (95% CI 1.43–4.38) for former users, a reversal of 48.0% from that for current users (meta-RR 4.81, 95% CI 2.05–11.30) of BQ-T, with overlapping 95% confidence intervals (***Table 1***, ***Figure 2***). Two studies reported results by duration of cessation [9,10]. While the reported categories were incompatible for a meta-analysis of the two studies, both studies reported a monotonic decrease of relative risks by duration since cessation.

However, the analysis of studies on BQ-T and oesophageal cancer did not reveal a substantial difference between the risk estimate for current and former users (***Table 1*** & Supplementary Figure 3).

### II. BQ+T cessation and risk of cancer

All the seven studies in this group were conducted in India: two cohort studies [13,14] and four case-control studies [15,16,17,18]. One study, a case-control nested in a randomized screening trial is considered together with the cohort studies because of the observational nature of cessation [19]. Six of the seven studies were restricted to men or provided sex-specific results [14,15,16,17,18,19]. Four studies did not provide a clear definition of former users [13,14,15,16]. Two case-control studies provided relative risks of oral cancer in former users of BQ+T by duration since cessation [15,16].

Majority of the studies (five of seven) adjusted for tobacco smoking and alcohol consumption as potential confounders or included only non-smokers and non-drinkers (Supplementary Table 3).

#### Quantitative analysis

**Figure 3:**
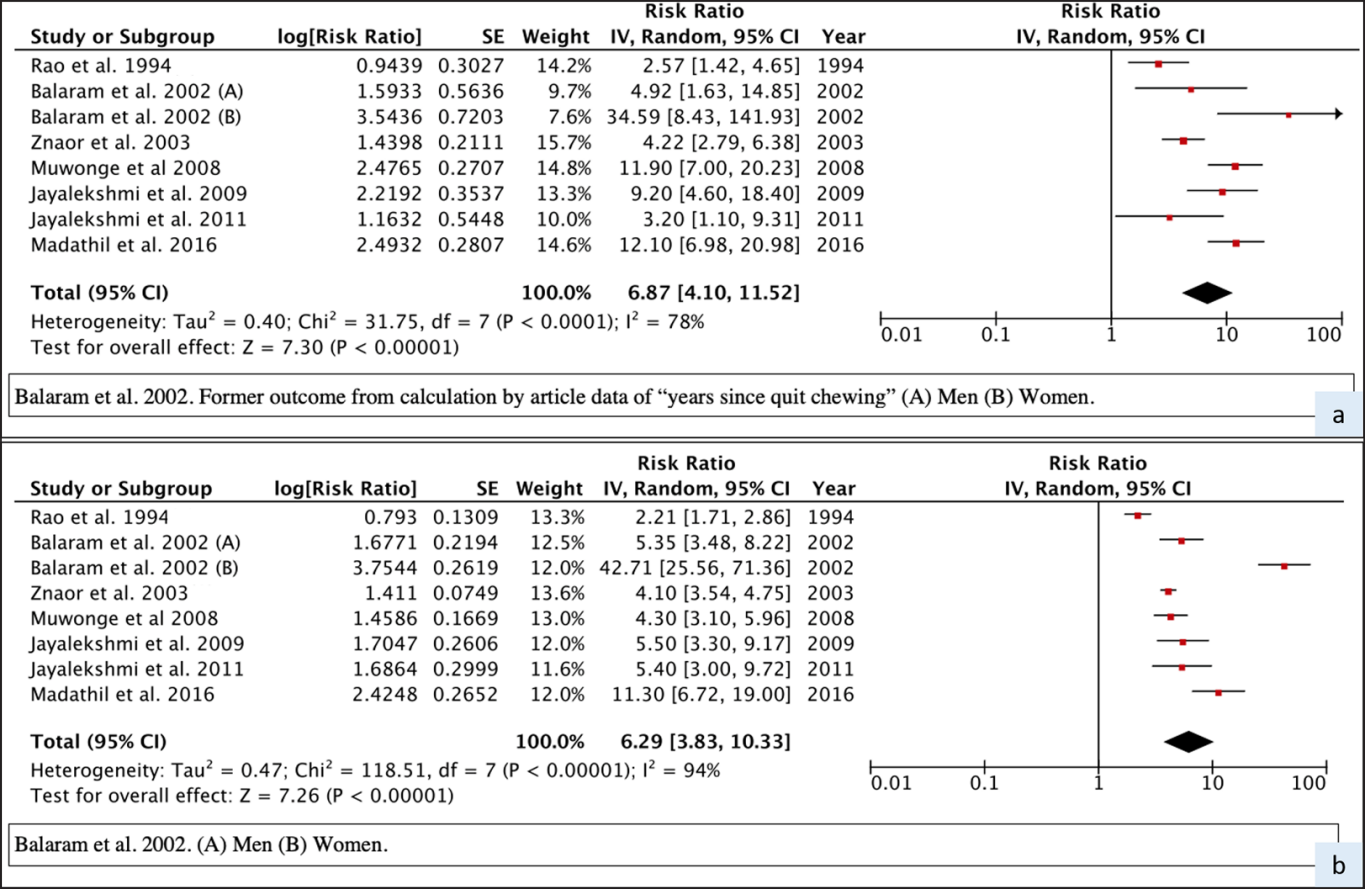
Forest plot and meta-RR (random effect) of the effect of BQ+T cessation on oral cancer risk (A – former users, B – current users).

The meta-relative risk (meta-RR) of oral cancer with all seven studies considered together was 6.29 (95% CI 3.83–10.33) for current BQ+T users and 6.87 (95% CI 4.10–11.52) for former users, depicted in ***Figure 3***. Restricting the analysis to studies that adjusted for the main potential confounders (tobacco smoking and alcohol drinking) gave the meta-RR for current users as 7.76 (95% CI 4.27–14.10) and former users as 9.02 (95% CI 5.32–15.29), shown in Supplementary Figure 4. Sub-group analysis by gender revealed in general substantially higher risks among women, but with a similar pattern of results with higher risk in former users (***Table 2***, Supplementary Figure 5).

**Table 2:** Summary risk estimates of studies evaluating the risk of oral cancer in current and former BQ+T users.


		STUDIES INCLUDED	PRODUCT AND TYPE OF USERS	SUMMARY RISK RATIO	95% CI

**Overall**		Ref 13–19	Current BQ+T user	6.29	3.83–10.33

			Former BQ+T user	6.87	4.10–11.52

**By Gender**	Males	Ref 14–17, 19	Current BQ+T user	3.60	2.55–5.08

			Former BQ+T user	3.96	2.99–5.25

	Females	Ref 14,15,19	Current BQ+T user	13.11	3.64–47.22

			Former BQ+T user	21.29	7.41–61-17

**Studies with cofounder adjustment**		Ref 13,15,16,18,19	Current BQ+T user	6.29	3.83–10.33

			Former BQ+T user	9.02	5.32–15.29

**Dropping cohort studies (problematic definition of “cessation”)**		15-18	Current BQ+T user	7.27	3.40-15.50

			Former BQ+T user	6.53	3.16–13.49

**Case control studies with confounder adjustment and reasonable definition of “former” user**		Ref 15,16	Current BQ+T user	9.61	2.77–33.30

			Former BQ+T user	7.63	2.57–22.63

**Duration of cessation**		Ref 15,16	Former user <10 years	1.21	0.90–1.63

			Former user >10 years	0.72	0.48–1.07


**Figure 4:**
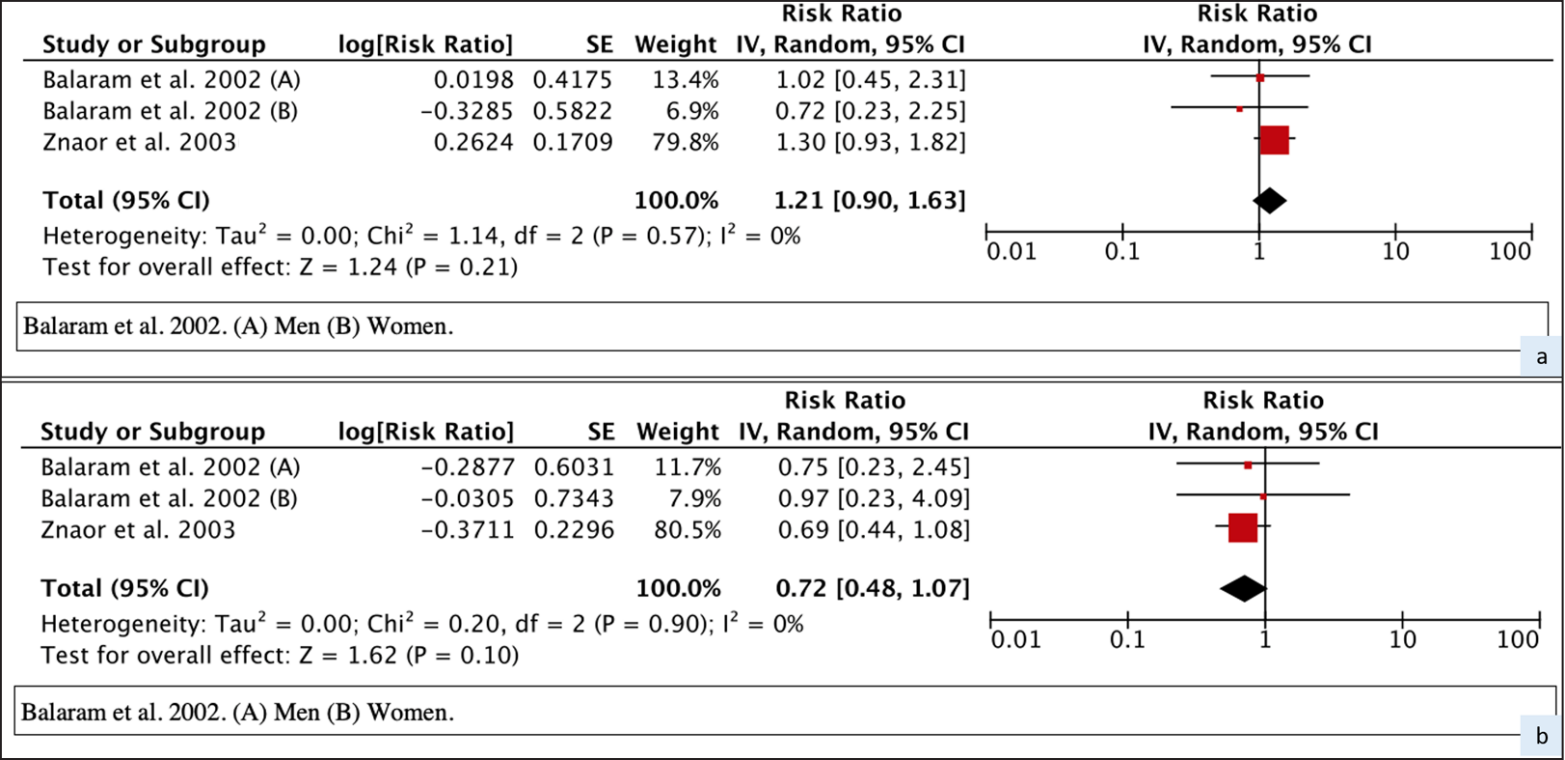
Forest plot and meta-RR (random effect) of the effect of BQ+T cessation duration on Oral cancer. (**A)** Former users <10yrs and (**B)** Former users >10yrs.

When the cohort studies with a problematic definition of former users were excluded, the meta-RR for current users was 7.27 (95% CI 3.40–15.50) while that for former users was 6.53 (95% CI 3.16–13.49). Further restriction to fully adjusted case-control studies with a clear definition of former users of BQ+T gave a meta-RR of current users as 9.61 (95% CI 2.77–33.30) and former users as 7.63 (95% CI 2.57–22.63), seen in Supplementary Figure 6. In contrast to the overall meta-analysis, these two sensitivity analyses successively dropping lower quality studies indicated a risk reversal in former users, but 95% confidence intervals were wide and overlapped. The two studies of the final sensitivity analysis also provided relative risks by duration since cessation with current users as the reference group. Meta-RR of former users who quit less than 10 years ago was 1.21 (95% CI 0.90–1.63) and 0.72 (95% CI 0.48–1.07) for those who quit more than 10 years ago (***Figure 4***) compared to the current users. Results restricted to men were very similar (data not shown). Visual inspection of the forest plots by duration since cessation did not indicate critical heterogeneity.

Only one study reported results on the effect of cessation of BQ+T use on cancers of the pharynx and oesophagus [16]. Compared to current users most of the relative risks by duration since cessation of BQ+T were below unity.

## Discussion

Based on the evidence of carcinogenicity of AN (with or without tobacco) from case-control, cohort studies in man and *in vivo* and *in vitro* experimental studies, the IARC Monographs have categorized AN, betel quid without added tobacco (BQ-T) and with added tobacco (BQ+T) as carcinogenic to humans (Group I) [2,5]. However, there is a paucity of data on the effect of cessation of this habit on the associated cancer risk. This situation is in stark contrast to that with tobacco smoking where several studies as well as meta-analyses have demonstrated a reduction in cancer risk after smoking cessation [2,20,21,22]. There is strong evidence of the reduction of the risk of lung cancer in those who quit early in their life approaching that of lifelong non-smokers [2,20,21]. Likewise, the risk other cancer sites causally linked to tobacco smoking has been shown to be significantly lower among ex-smokers than that of current smokers, typically after about ten years since quitting, with some variation across cancer sites and histologies [20,23]. For example, for oesophageal carcinoma, after 10 years of smoking abstinence, former smokers still harbour a risk of squamous-cell oesophageal cancer which is twice than that of never smokers, and for adenocarcinoma of the oesophagus some decline in risk, if any, was suggested only after 20–30 years of cessation [20].

With respect to AN, the most recent IARC Monograph discussed effects of cessation only for oral cancer and concluded that “the effect of cessation has not been examined extensively” [2,5]. The present SRMA is, to the best of our knowledge, the first attempt at summarising the available evidence of the effect of BQ-T cessation on the risk of oral, pharyngeal and oesophageal cancers and BQ+T quitting on oral cancer. The fact that only 14 studies (seven each for BQ-T and BQ+T) could be identified for this analysis demonstrates that there still is limited research on this important aspect of AN abuse. Our analysis showed a 28.9% reversal in the oral cancer risk after cessation of BQ-T habit while the risk of pharyngeal cancer dropped by 48.0% in former users of BQ-T compared to the current users. All four studies on oral cancer adjusted for tobacco smoking as a confounder while three studies [6,8,9] also adjusted for alcohol consumption. All three studies on pharyngeal cancer adjusted for tobacco smoking and alcohol consumption as confounders.

Together, these results support the potential benefit of BQ-T cessation on risk of oral and pharyngeal cancers. However, the accumulated evidence is based only on a few studies and confidence intervals of meta-RR overlap between current and former users. Only one study on oral cancer and two studies on pharyngeal cancer reported data by duration of cessation. While these studies support the risk reversal after cessation, a well-characterized timeline of cancer risk patterns following BQ-T or AN cessation remains to be determined.

The four studies on oral cancer included in the SRMA showed important heterogeneity (I^2^ > 50%), though the funnel plot for the same demonstrates symmetry indicating low publication bias.

For oesophageal cancer, no substantial difference was noted in the risk between current and former users of BQ-T in the present meta-analysis. One of the two original studies reported risks for current and former BQ-T chewers by location [12]. However, the study results were not sufficiently precise to delineate a specific pattern by location. Studies on tobacco smoking and oesophageal cancer have identified patterns of risk reversal by histology, in that squamous cell carcinoma showed a strong risk reduction after smoking cessation for more than 10 years while adenocarcinoma demonstrated only a slight risk reduction among former smokers compared to current smokers [20,24]. Both studies on cessation of BQ-T use included only histologically verified cases of squamous cell carcinomas.

For BQ+T cessation, overall meta-relative risk for oral cancer seemed to be higher among former users than current users. This may partly be explained by methodical issues in the original studies, particularly regarding the definition and assessment of cessation of use and the relative low proportion of long-term quitters among former users. The sensitivity analysis of the higher quality case control studies revealed a small reduction in the risk of oral cancer among former users of BQ+T. Further, in the same subset of studies, the risk of oral cancer among persons who quit less than 10 years ago was higher than in current users, but after more than 10 years of cessation the meta-RR was lower than among current chewers. Hence, our meta-analysis supports the notion that cessation of BQ+T chewing habit has a beneficial effect on oral cancer risk, though this may become apparent only after more than 10 years of cessation. However, the confidence intervals of meta-RR in this analysis between short-term and long-term quitters were overlapping. Hence, the evidence provided by this SRMA may be considered as indicative, requiring further well-designed studies.

The main strengths of the present meta-analysis include the first-of-its-kind summarization of the available evidence of the effect of BQ cessation on all upper digestive tract cancers causally linked to AN use, with a priori stratification by type of BQ (BQ-T vs BQ+T) use and careful assessment of the validity of the original studies, particularly with regards to the assessment of cessation of habits. The time-dependent analysis of the cancer risk reversal after BQ+T cessation is coherent with the more robust evidence on risk reversal after cessation of tobacco smoking. However, the analysis also suffered from a few limitations, the most important being the few numbers of studies that provided separate risk estimates for current and former chewers, and even fewer of the better-quality studies reported results by duration since cessation. The studies included in the SRMA did not adjust for the intensity and duration of BQ chewing among current or former users. Given the strong effect of BQ use on the selected cancer sites and a plausible association between these metrics of BQ use, there is potential for bias. However, the magnitude of this potential bias is difficult to assess in the absence of quantitative data. Further, there are only little data on risk reversal among women. Given the higher cancer risks in female users of BQ this is an important gap. The confidence intervals of meta-RRs in most of the analyses overlapped between current and former users indicating the need of additional valid and precise analytical epidemiological studies on the cessation of BQ use.

## Conclusion

This first-of-its-kind systematic review and meta-analysis provides initial evidence on the reduction of risk of upper digestive tract cancers after cessation of betel quid consumption. The currently available data also demonstrate a persisting increased cancer risk in those who quit this habit compared to never chewers, indicating that careful surveillance is warranted even after quitting of BQ use. For BQ+T, the reduction in risk of cancer only started to show in long-term quitters (>10 years). Future research needs to enrol sizable numbers of women quitters to allow for sex-specific analysis by type of BQ chewed, with clear definition and assessment of cessation, adjust analyses for the major potential confounders (including intensity and duration of BQ use), and large numbers of long-term quitters to refine the evidence on risk reversal by time since quitting. Pooled re-analyses of published studies on BQ use and cancer risk may be an efficient research strategy. There is need to educate the public and health professionals of the addictive and carcinogenic effects of betel quid – both with and without tobacco – and to develop culturally sensitive models on BQ cessation. Our results provide additional evidence to policy makers and various stakeholders to support and promote primary prevention including cessation of BQ chewing more vigorously.

## Additional File

The additional file for this article can be found as follows:

10.5334/aogh.3643.s1 Supplementary File. Supplementary Tables and Figures.
