# Supplementary material for: Risk Reversal of Oral, Pharyngeal and Oesophageal Cancers after Cessation of Betel Quid Users: A Systematic Review and Meta-Analysis
Source: Ann Glob Health. 2022 Jan 11;88(1):5. doi: 10.5334/aogh.3643 (PMC8757388; doi:10.5334/aogh.3643)
Supplement: Supplementary File. — Supplementary Tables and Figures. [file agh-88-1-3643-s1.pdf]

Supplementary Table 1 Search terms used in the present systematic review and meta-analysis#

**For Areca nut**		
Betel nut OR areca nut OR betel quid OR betel leaf OR betel inflorescence OR Paan OR pan OR supari		
**For Oral Cancer**	**For Pharyngeal cancer**	**For Esophageal cancer**
oral OR buccal OR oral cavity OR lip OR labial OR tongue OR base tongue OR anterior tongue OR posterior tongue OR gingiva OR cheek OR floor of mouth OR palate	pharyng* OR orophrayn* OR hypopharyng*	oesophag* OR esophag*

# Search terms combined the ‘Areca nut’ and one of the cancer sites

Supplementary Table 2 Characteristics of studies on betel quid without added tobacco (BQ-T) included in the systematic review and meta-analysis

Author/ year	Country	Study design	Number of cases/controls	Type of user	OR (95% CI)	Adjustment for potential confounders
**ORAL CANCER**						
Ko 1995	Taiwan	Case control	71 / 42	Current	6.90 (3.10-15.20)	Education, occupation, alcohol, smoking
			5 / 5	Former	4.70 (0.90-22.70)	
Thomas 2007	Papua New Guinea	Case control	124 / 375	Current	1.29 (0.25-6.51)	Age, sex, province, residence, income, education, frequency of smoking
			9 / 56	Former	0.57 (0.10-3.28)	
Lee 2012	Taiwan	Case control	450 / 160	Current	16.70 (12.10-23.0)	Gender, age, ethnicity, education, alcohol intake, smoking, consumption of vegetables and fruits
			224 / 88	Former	15.30 (10.60-22.0)	
Wu 2016	Taiwan	Case control	113 / 66	Current	8.05 (5.10-12.71)	Age, education, cigarette smoking (pack-year categories), alcohol drinking (frequency)
			133 / 105	Former	6.43 (4.25-9.73)	
**PHARYNGEAL CANCER**						
Lee 2012	Taiwan	Case control	147 /160	Current	9.3 (6.1-14.2)	Gender, age, ethnicity, education, alcohol intake, cigarette smoking, consumption of vegetables & fruits
			29 / 88	Former	3.5 (2-6.1)	
Wu 2016	Taiwan	Case control	45 / 66	Current	4.8 (2.57-8.9)	Age, education, cigarette smoking, alcohol drinking
			43 / 105	Former	2.87 (1.61-5.13)	
Zeng 2019	China	Case control	63 / 73	Current	2.52 (1.73-3.67)	BMI, income, smoking habits, alcohol drinking, oral hygiene
			18 / 43	Former	1.13 (0.44-2.87)	
**OESOPHAGEAL CANCER**						
Lee 2007	Taiwan	Case control	30/ 46 upper	Current	4.90 (2.50-9.50)	Gender, age, ethnicity, education, drink-years of alcohol, pack-years of smoking and consumption of vegetables and fruits
			44 / 46 middle		2.20 (1.30-3.80)	
			36 / 46 lower		3.70 (2.0-6.80)	
			27 / 41 upper	Former	4.60 (2.30-9.0)	

			47 / 41 middle		2.20 (1.30-3.90)	
			26 / 41 lower		2.80 (1.50-5.30)	
Wu 2006	Taiwan	Case control	62 / 28	Current	1.6 (0.8-3.3)	Cigarette smoking, alcohol, drinking, age, and years of education
			31 / 11	Former	1.8 (0.8-4.6)	

Supplementary Table 3 Study characteristics of the included studies on BQ+T chewing and oral cancer

Author/ year	Country	Study design	Sample size (cases/controls or cohort)	Type of user	OR (95% CI)	Confounders adjusted
Rao 1994	India	Case control	279/ 193	Current	2.21 (1.71-2.92)	None
			124/ 24	Former <1 yr	5.82 (3.64-10.21)	
			42/ 15	Former >1 yr	2.57 (1.42-5.77)	
Balaram 2002	India	Case control	247/ 269	Current (men)	5.35 (3.48-8.22)	Age, education, smoking, drinking
			232/ 280	Current (women)	42.71 (25.56-71.36)	
			59/ 20	Former (men)	4.92 (1.63-14.85)	
			48/ 9	Former (women)	34.59 (8.43-141.93)	
			45/ 14	Former <10 yr (men)	1.02 (0.45-2.29)	
			31/ 6	Former <10 yr (women)	0.72 (0.23-2.25)	
			14/ 6	Former >10 yr (men)	0.75 (0.23-2.52)	
			17/ 3	Former >10 yr (women)	0.97 (0.23-4.09)	
Znaor 2003	India	Case control	160/ 445	Current	4.30 (3.10-6.10)	Age, center, education level, alcohol consumption and smoking
			212/ 99	Former	9.27 (7.21-11.93)	
			152/ 61	Former <10 yr	1.30 (0.93-1.82)	
			60/38	Former >10 yr	0.69 (0.44-1.08)	

Muwonge 2008	India	Case control	160/445	Current	4.30 (3.10-6.10)	Education, religion, smoking, alcohol
			42/ 50	Former	11.90 (7.00-20.4)	
Jayaleksh mi 2009	India	Cohort	183749 PY	Current	5.50 (3.30-9.00)	Age, income, education, smoking, alcohol
			26804 PY	Former	9.20 (4.60-18.10)	
			13817 PY	Former <10yr	14.32 (5.75-32.09)	
			4819 PY	Former >10yr	23.47 (6.98-62.86)	
Jayaleksh mi 2011	India	Cohort	218673 PY	Current	2.40 (1.70-3.30)	Age, income, education
			49079 PY	Former	2.10 (1.30-3.60)	
Madathil 2016	India	Case control	131/36	Current	11.3 (6.72-19.05)	Age, sex, pack-years of bidi, cigarette smoking, alcohol, number of missing teeth, lifetime material deprivation index, & weekly vegetable consumption
			109/26	Former	12.1 (6.98-21.01)	

PY: person years; Yr: years

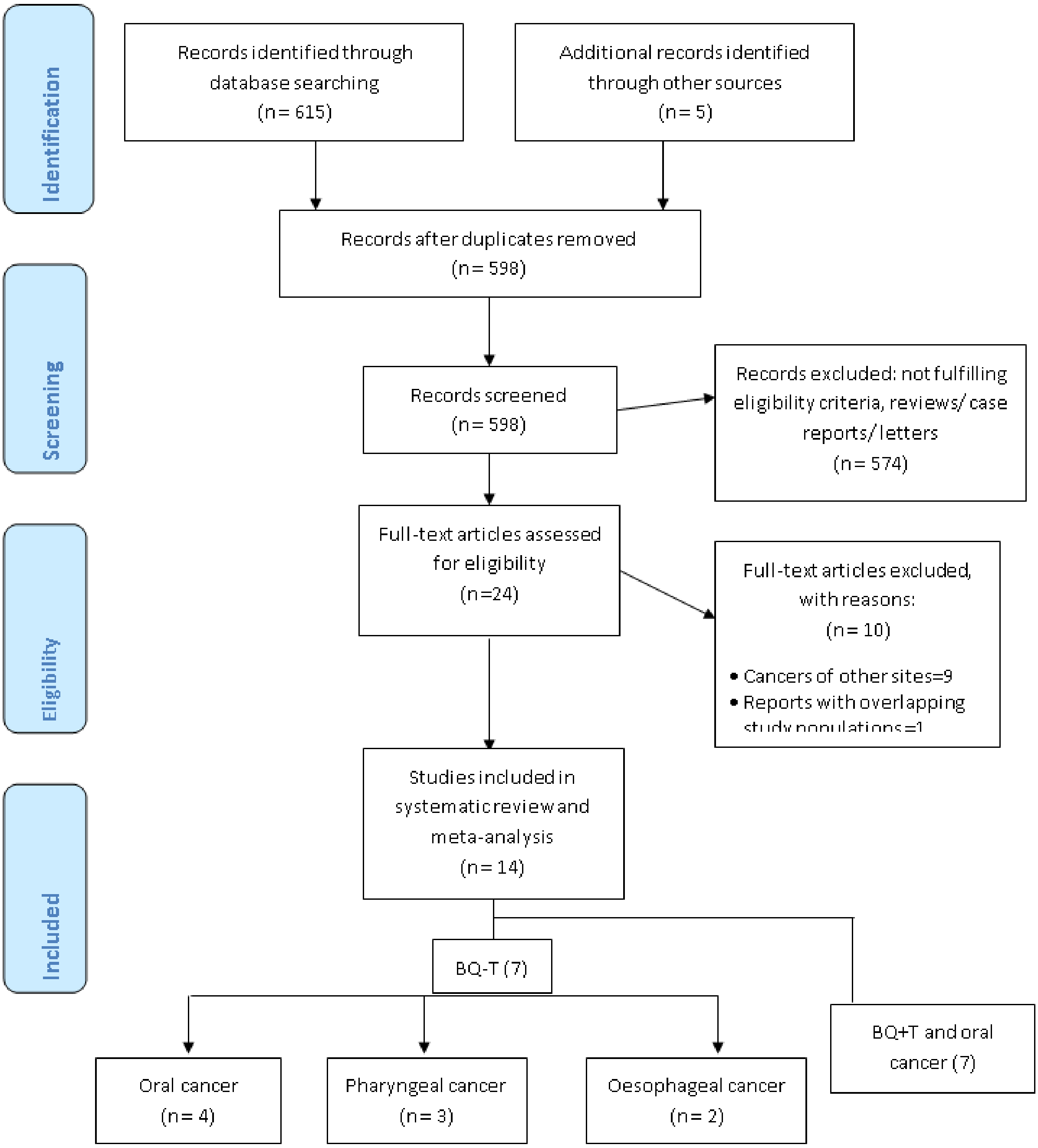

Supplementary Figure 1 PRISMA flow diagram from study identification to inclusion for meta-analysis

A

Study or Subgroup	log[Risk Ratio]	SE	Weight	Risk Ratio IV, Random, 95% CI
Ko et al. 1995	1.5476	0.8433	14.5%	4.70 [0.90, 24.54]
Lee et al. 2012	2.7279	0.1872	43.3%	15.30 [10.60, 22.08]
Thomas et al. 2007	-0.5621	0.888	0.0%	0.57 [0.10, 3.25]
Wu et al. 2016	1.861	0.2113	42.1%	6.43 [4.25, 9.73]
**Total (95% CI)**			**100.0%**	**8.95 [4.21, 19.00]**

Heterogeneity: $Tau^2 = 0.31$; $Chi^2 = 10.30$, df = 2 (P = 0.006); $I^2 = 81\%$
Test for overall effect: Z = 5.70 (P < 0.00001)

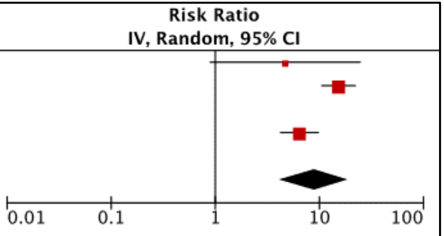

B

Study or Subgroup	log[Risk Ratio]	SE	Weight	Risk Ratio IV, Random, 95% CI
Ko et al. 1995	1.9315	0.4082	24.8%	6.90 [3.10, 15.36]
Lee et al. 2012	2.8154	0.1644	39.7%	16.70 [12.10, 23.05]
Thomas et al. 2007	0.2546	0.8372	0.0%	1.29 [0.25, 6.66]
Wu et al. 2016	2.0857	0.2329	35.5%	8.05 [5.10, 12.71]
**Total (95% CI)**			**100.0%**	**10.35 [5.71, 18.77]**

Heterogeneity: $Tau^2 = 0.21$; $Chi^2 = 8.78$, df = 2 (P = 0.01); $I^2 = 77\%$
Test for overall effect: Z = 7.70 (P < 0.00001)

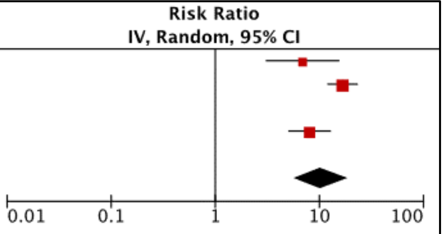

Supplementary Figure 2 Forest plot and meta-RR (random effect) of the effect of BQ-T cessation on oral cancer risk, sensitivity analysis (A – former users, B – current users)

A

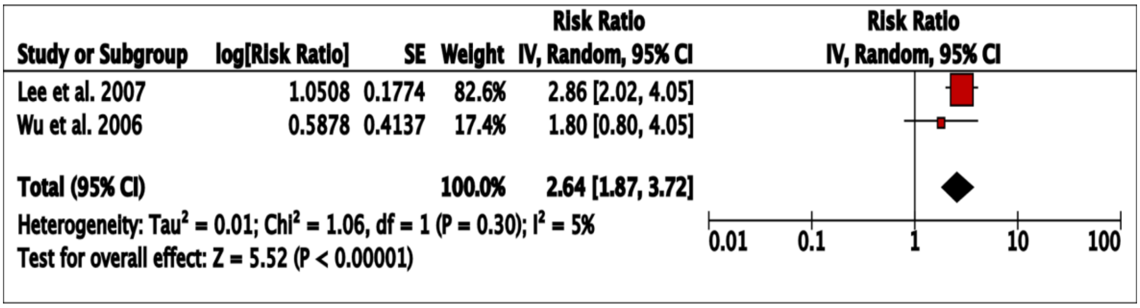

B

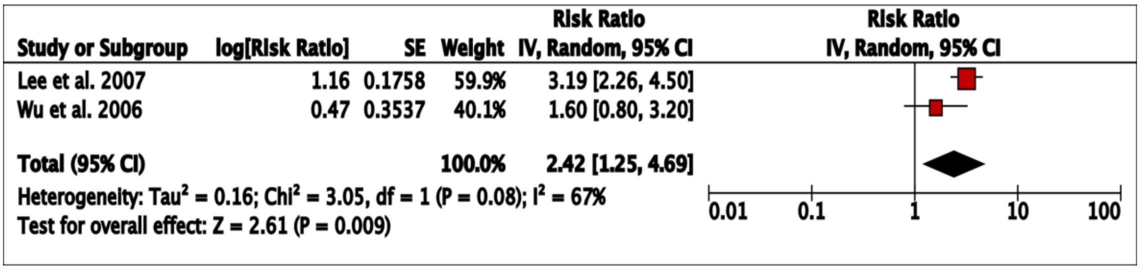

Supplementary Figure 3 Forest plot and meta-RR (random effect) of the effect of BQ-T cessation on oesophageal cancer risk (A – former users, B – current users)

A

Study or Subgroup	log[Risk Ratio]	SE	Weight	Risk Ratio IV, Random, 95% CI	Year
Rao et al. 1994	0.9439	0.3027	0.0%	2.57 [1.42, 4.65]	1994
Balaram et al. 2002 (A)	1.5933	0.5636	12.0%	4.92 [1.63, 14.85]	2002
Balaram et al. 2002 (B)	3.5436	0.7203	9.0%	34.59 [8.43, 141.93]	2002
Znaor et al. 2003	1.4398	0.2111	21.8%	4.22 [2.79, 6.38]	2003
Muwonge et al 2008	2.4765	0.2707	20.0%	11.90 [7.00, 20.23]	2008
Jayalekshmi et al. 2009	2.2192	0.3537	17.5%	9.20 [4.60, 18.40]	2009
Jayalekshmi et al. 2011	1.1632	0.5448	0.0%	3.20 [1.10, 9.31]	2011
Madathil et al. 2016	2.4932	0.2807	19.7%	12.10 [6.98, 20.98]	2016
**Total (95% CI)**			**100.0%**	**9.02 [5.32, 15.29]**	

Heterogeneity: $Tau^2 = 0.29$; $Chi^2 = 18.51$, df = 5 (P = 0.002); $I^2 = 73\%$
Test for overall effect: Z = 8.17 (P < 0.00001)

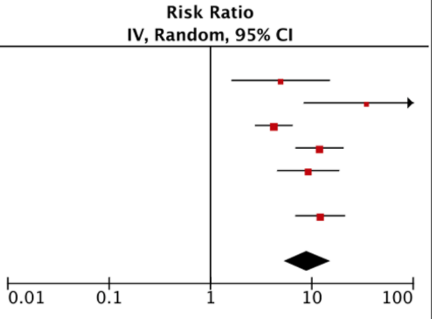

Balaram et al. 2002. Former outcome from calculation by article data of "years since quit chewing" (A) Men (B) Women.

B

Study or Subgroup	log[Risk Ratio]	SE	Weight	Risk Ratio IV, Random, 95% CI	Year
Rao et al. 1994	0.793	0.1309	0.0%	2.21 [1.71, 2.86]	1994
Balaram et al. 2002 (A)	1.6771	0.2194	16.6%	5.35 [3.48, 8.22]	2002
Balaram et al. 2002 (B)	3.7544	0.2619	16.0%	42.71 [25.56, 71.36]	2002
Znaor et al. 2003	1.411	0.0749	18.0%	4.10 [3.54, 4.75]	2003
Muwonge et al 2008	1.4586	0.1669	17.3%	4.30 [3.10, 5.96]	2008
Jayalekshmi et al. 2009	1.7047	0.2606	16.1%	5.50 [3.30, 9.17]	2009
Jayalekshmi et al. 2011	1.6864	0.2999	0.0%	5.40 [3.00, 9.72]	2011
Madathil et al. 2016	2.4248	0.2652	16.0%	11.30 [6.72, 19.00]	2016
**Total (95% CI)**			**100.0%**	**7.76 [4.27, 14.10]**	

Heterogeneity: $Tau^2 = 0.51$; $Chi^2 = 84.51$, df = 5 (P < 0.00001); $I^2 = 94\%$
Test for overall effect: Z = 6.72 (P < 0.00001)

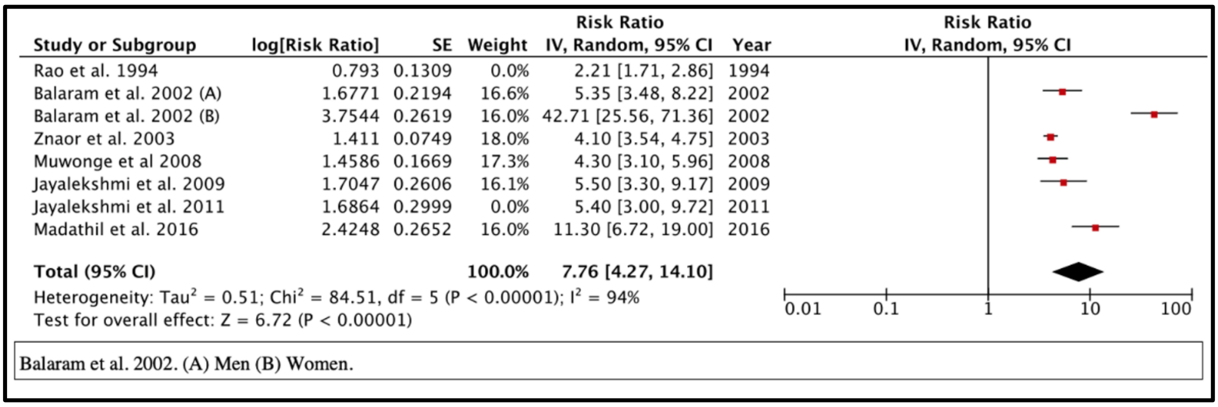

Balaram et al. 2002. (A) Men (B) Women.

Supplementary Figure 4 Forest plot and meta-RR (random effect) of the effect of BQ+T cessation on Oral cancer risk for former users (A) and (B) current users adjusted for potential confounders

A

Study or Subgroup	log[Risk Ratio]	SE	Weight	Risk Ratio IV, Random, 95% CI	Year
Rao et al. 1994	0.9439	0.3027	22.6%	2.57 [1.42, 4.65]	1994
Balaram et al. 2002	1.5933	0.5636	6.5%	4.92 [1.63, 14.85]	2002
Znaor et al. 2003	1.4398	0.2111	46.5%	4.22 [2.79, 6.38]	2003
Muwonge et al 2008	1.775	0.3451	17.4%	5.90 [3.00, 11.60]	2008
Jayalekshmi et al. 2011	1.1632	0.5448	7.0%	3.20 [1.10, 9.31]	2011
**Total (95% CI)**			**100.0%**	**3.96 [2.99, 5.25]**	

Heterogeneity: $Tau^2 = 0.00$; $Chi^2 = 3.77$, df = 4 ($P = 0.44$); $I^2 = 0\%$
Test for overall effect: $Z = 9.56$ ($P < 0.00001$)

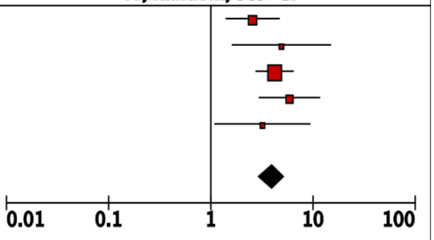

B

Study or Subgroup	log[Risk Ratio]	SE	Weight	Risk Ratio IV, Random, 95% CI
Balaram et al. 2002	1.6771	0.2194	18.5%	5.35 [3.48, 8.22]
Jayalekshmi et al. 2011	1.6864	0.2999	14.8%	5.40 [3.00, 9.72]
Muwonge et al 2008	0.9933	0.2069	19.1%	2.70 [1.80, 4.05]
Rao et al. 1994	0.793	0.1309	22.7%	2.21 [1.71, 2.86]
Znaor et al. 2003	1.411	0.0749	24.8%	4.10 [3.54, 4.75]
**Total (95% CI)**			**100.0%**	**3.60 [2.55, 5.08]**

Heterogeneity: $Tau^2 = 0.12$; $Chi^2 = 23.93$, df = 4 ($P < 0.0001$); $I^2 = 83\%$
Test for overall effect: $Z = 7.28$ ($P < 0.00001$)

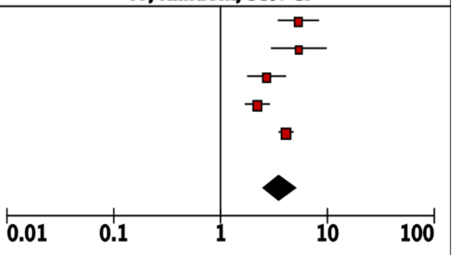

C

Study or Subgroup	log[Risk Ratio]	SE	Weight	Risk Ratio IV, Random, 95% CI
Balaram et al. 2002	3.5436	0.7203	25.8%	34.59 [8.43, 141.93]
Jayalekshmi et al. 2009	2.2192	0.3537	39.8%	9.20 [4.60, 18.40]
Muwonge et al 2008	3.6636	0.4875	34.4%	39.00 [15.00, 101.40]
**Total (95% CI)**			**100.0%**	**21.29 [7.41, 61.17]**

Heterogeneity: $Tau^2 = 0.60$; $Chi^2 = 6.89$, df = 2 ($P = 0.03$); $I^2 = 71\%$
Test for overall effect: $Z = 5.68$ ($P < 0.00001$)

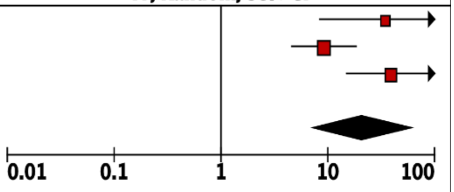

D

Study or Subgroup	log[Risk Ratio]	SE	Weight	Risk Ratio IV, Random, 95% CI
Balaram et al. 2002	3.7544	0.2619	33.7%	42.71 [25.56, 71.36]
Jayalekshmi et al. 2009	1.7047	0.2606	33.7%	5.50 [3.30, 9.17]
Muwonge et al 2008	2.2513	0.3275	32.7%	9.50 [5.00, 18.05]
**Total (95% CI)**			**100.0%**	**13.11 [3.64, 47.22]**

Heterogeneity: $Tau^2 = 1.20$; $Chi^2 = 32.36$, df = 2 ($P < 0.00001$); $I^2 = 94\%$
Test for overall effect: $Z = 3.93$ ($P < 0.0001$)

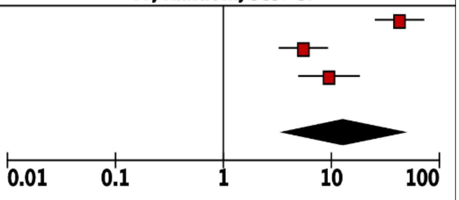

Supplementary Figure 5 Forest plot and meta-RR (random effect) of the effect of BQ+T cessation on Oral cancer risk by gender. (A) Former men users and (B) current men users (C) former women users and (D) current women users.

A

Study or Subgroup	log[Risk Ratio]	SE	Weight	Risk Ratio IV, Random, 95% CI	Year
Rao et al. 1994	0.9439	0.3027	0.0%	2.57 [1.42, 4.65]	1994
Balaram et al. 2002 (A)	1.5933	0.5636	31.1%	4.92 [1.63, 14.85]	2002
Balaram et al. 2002 (B)	3.5436	0.7203	25.9%	34.59 [8.43, 141.93]	2002
Znaor et al. 2003	1.4398	0.2111	43.0%	4.22 [2.79, 6.38]	2003
Muwonge et al 2008	2.4765	0.2707	0.0%	11.90 [7.00, 20.23]	2008
Jayalekshmi et al. 2009	2.2192	0.3537	0.0%	9.20 [4.60, 18.40]	2009
Jayalekshmi et al. 2011	1.1632	0.5448	0.0%	3.20 [1.10, 9.31]	2011
Madathil et al. 2016	2.4932	0.2807	0.0%	12.10 [6.98, 20.98]	2016
**Total (95% CI)**			**100.0%**	**7.63 [2.57, 22.63]**	

Heterogeneity: $Tau^2 = 0.67$; $Chi^2 = 7.86$, df = 2 (P = 0.02); $I^2 = 75\%$
Test for overall effect: Z = 3.66 (P = 0.0003)

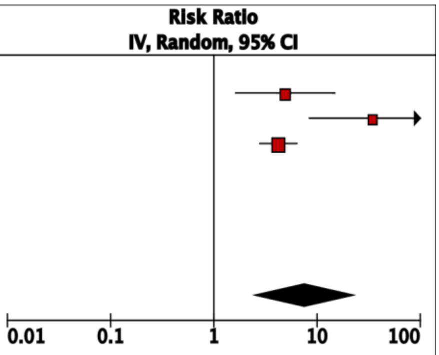

Balaram 2002: (A) Men; (B) Women

B

Study or Subgroup	log[Risk Ratio]	SE	Weight	Risk Ratio IV, Random, 95% CI	Year
Rao et al. 1994	0.793	0.1309	0.0%	2.21 [1.71, 2.86]	1994
Balaram et al. 2002 (A)	1.6771	0.2194	33.1%	5.35 [3.48, 8.22]	2002
Balaram et al. 2002 (B)	3.7544	0.2619	32.6%	42.71 [25.56, 71.36]	2002
Znaor et al. 2003	1.411	0.0749	34.3%	4.10 [3.54, 4.75]	2003
Muwonge et al 2008	1.4586	0.1669	0.0%	4.30 [3.10, 5.96]	2008
Jayalekshmi et al. 2009	1.7047	0.2606	0.0%	5.50 [3.30, 9.17]	2009
Jayalekshmi et al. 2011	1.6864	0.2999	0.0%	5.40 [3.00, 9.72]	2011
Madathil et al. 2016	2.4248	0.2652	0.0%	11.30 [6.72, 19.00]	2016
**Total (95% CI)**			**100.0%**	**9.61 [2.77, 33.30]**	

Heterogeneity: $Tau^2 = 1.17$; $Chi^2 = 74.16$, df = 2 (P < 0.00001); $I^2 = 97\%$
Test for overall effect: Z = 3.57 (P = 0.0004)

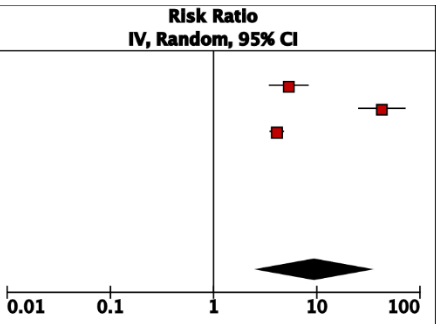

Balaram 2002: (A) Men; (B) Women

Supplementary Figure 6 Forest plot and meta-RR (random effect) of the effect of BQ+T cessation on Oral cancer risk in former (A) and current (B) users adjusted for potential confounders and providing reasonable definition of cessation.
